# CaMKII-Mediated CREB Phosphorylation Is Involved in Ca^2+^-Induced BDNF mRNA Transcription and Neurite Outgrowth Promoted by Electrical Stimulation

**DOI:** 10.1371/journal.pone.0162784

**Published:** 2016-09-09

**Authors:** Xiaodong Yan, Juanfang Liu, Zhengxu Ye, Jinghui Huang, Fei He, Wei Xiao, Xueyu Hu, Zhuojing Luo

**Affiliations:** 1 Department of Orthopaedics, Xijing Hospital, Fourth Military Medical University, Xi’an 710032, China; 2 Department of Orthopaedics, Tangdu Hospital, Fourth Military Medical University, Xi’an 710038, China; 3 Department of Clinical Aerospace Medicine, Fourth Military Medical University, Xi’an 710032, China; 4 Department of Hereditary and Development, Basic Unit, Fourth Military Medical University, Xi’an 710032, China; University of Louisville, UNITED STATES

## Abstract

Electrical stimulation (ES)-triggered up-regulation of brain-derived neurotrophic factor (BDNF) and neurite outgrowth in cultured rat postnatal dorsal root ganglion neurons (DRGNs) is calcium (Ca^2+^)-dependent. The effects of increased Ca^2+^ on BDNF up-regulation and neurite outgrowth remain unclear. We showed here that ES increased phosphorylation of the cAMP-response element binding protein (CREB). Blockade of Ca^2+^ suppressed CREB phosphorylation and neurite outgrowth. Down-regulation of phosphorylated (p)-CREB reduced BDNF transcription and neurite outgrowth triggered by ES. Furthermore, blockade of calmodulin-dependent protein kinase II (CaMKII) using the inhibitors KN93 or KN62 reduced p-CREB, and specific knockdown of the CaMKIIα or CaMKIIβ subunit was sufficient to suppress p-CREB. Recombinant BDNF or hyperforin reversed the effects of Ca^2+^ blockade and CaMKII knockdown. Taken together, these data establish a potential signaling pathway of Ca^2+^-CaMKII-CREB in neuronal activation. To our knowledge, this is the first report of the mechanisms of Ca^2+^-dependent BDNF transcription and neurite outgrowth triggered by ES. These findings might help further investigation of complex molecular signaling networks in ES-triggered nerve regeneration *in vivo*.

## Introduction

Electrical stimulation (ES) induces regeneration-related gene expression and neurite outgrowth of dorsal root ganglion neurons (DRGNs) [1, 2], and accelerates axon regeneration of central and peripheral nerves [3, 4]. Our previous study showed that ES accelerates neurite outgrowth and expression of brain-derived neurotrophic factor (BDNF), accompanied by an elevation in intracellular calcium (Ca^2+^) [5]. However, the correlation between Ca^2+^ increase and ES-induced neurite outgrowth and BDNF expression remains unclear.

One important neurotrophic factor, BDNF, mediates neuronal development and synaptic function [6], which is critical for differentiation and survival of neurons during development [7]. BDNF transcription is Ca^2+^-dependent [6, 8]. The cAMP-response element binding protein (CREB) mediates BDNF transcription [6]. A number of pathways that may contribute to CREB activation by phosphorylation at Ser-133 have been identified [6, 9–12]. Studies have shown that Ca^2+^, as an important messenger acting *via* Ca^2+^/calmodulin-dependent protein kinases (CaMKs), triggers phosphorylation of CREB [13], and that phosphorylated CREB (p-CREB) activates BDNF transcription by binding to a calcium response element within the gene [6].

The Ca^2+^ signaling that activates CREB can be traced back to CaMKs, which are activated by the Ca^2+^/calmodulin complex. Numerous Ca^2+^-evoked responses are regulated by calmodulin, a Ca^2+^-binding protein, and its downstream molecules CaMKs, such as CaMK-kinase (CaMKK), CaMKI, CaMKII, and CaMKIV [14]. Although CREB can be phosphorylated at the Ser-133 residue by all CaMKs, CaMKI and CaMKIV must be phosphorylated by a CaMK kinase before being activated by calcium and calmodulin [15]. CaMKI mediates Ca^2+^-induced neurite outgrowth through activation of extracellular signal-regulated kinase or microtubule affinity-regulating kinase 2 (MARK2), and CaMKIV mediates neuritogenesis by phosphorylation of CREB and subsequent CREB-dependent transcription [16]. It has been proposed that the CaMKK-CaMKIV cascade is involved in Ca^+^-induced neurite growth through CREB phosphorylation and transcriptional activation [11, 17]. CaMKII, which is encoded by four different genes, includes about 12 subunits [18, 19]. The CaMKII α and β subunits are abundant in nervous systems, whereas CaMKII γ and δ are widely present in non-neuronal tissues [20]. CaMKII can be activated by Ca^2+^ and calmodulin, resulting in autophosphorylation. CaMKII phosphorylation activates signaling molecules and several other transcription factors including CREB [13]. In addition, the CaMKIV-CREB pathway mediates dendrite growth in hippocampal neurons [11]. However, the contributions of Ca^2+^ increase and CaMKs to ES-induced BDNF transcription and neurite outgrowth have not previously been shown. Furthermore, although differential transcription of the BDNF gene is involved in a specific region of central and peripheral nervous systems [21, 22], the mechanisms by which BDNF transcription is up-regulated in cultured DRGNs by exogenic factors, such as ES, remain unclear.

In the present study, we investigated the role of CREB in ES-induced BDNF transcription and neurite outgrowth. We show that ES induced p-CREB, which was effectively suppressed by the application of Ca^2+^ inhibitors. Down-regulation of CaMKII through a pharmacological approach or RNA interference markedly reduced p-CREB. Application of Ca^2+^ inhibitors or knockdown of CREB or CaMKII reduced BDNF transcription and neurite outgrowth. Our data established a molecular pathway of Ca^2+^-CaMKII-CREB that can mediate ES-induced BDNF transcription and neurite outgrowth in cultured DRGNs.

## Materials and Methods

### Preparation of rat DRGN cultures

Culture of primary DRGNs was performed by following the procedures described previously [5]. DRGs (C5-L6) from neonatal 24 hr-old Sprague Dawley rats (Laboratory Animal Center of Fourth Military Medical University) were harvested and digested in a mixture of 0.05% collagenase IV (Sigma, St. Louis, MO) and 0.125% trypsin (Sigma, St. Louis, MO). Cells were cultured in Neurobasal medium A (Gibco BRL, Grand Island, NY) containing 1% penicillin and streptomycin, 2 mM Glutamax I (Gibco BRL), and 2% B27 supplement (Gibco BRL). Mouse anti-rat β-tubulin III monoclonal antibody (1:500; Chemicon, USA) was used to assess the purity of DRGN cultures by immunocytochemistry. The purity of β-tubulin III positive cells was higher than 92%, as determined by the described evaluation method [5]. Animal surgery and experimental procedures were approved by the Laboratory Animal Welfare and Ethics Committee of Fourth Military Medical University and were carried out according to the principles of laboratory animal care.

### Electrical stimulation of DRGNs

DRGNs were treated with ES using conductive indium tin oxide (ITO) conductive silica glass (30 Ω/cm^2^; Kinoene Kogaku, Japan) by following the protocol described previously [5]. Briefly, after 1-day growth at 37°C in 5% CO_2_, DRGNs cultured on the ITO glass were stimulated for 0–60 min with biphasic rectangular pulses delivered at 5 V and 10 Hz. Neurons in sham groups were cultured on the ITO glass without ES. Before ES application, neurons in treatment groups were incubated in culture medium supplemented with drugs at 37°C in 5% CO_2_ for 30 min.

### Immunocytochemistry assay for p-CREB in DRGNs

Neurons were first fixed with phosphate-buffered 4% paraformaldehyde (pH 7.35) at room temperature for 20 min. After wash with 0.01 M phosphate buffered saline (PBS) (pH 7.5), neurons were permeabilized in 0.1% Triton X-100 for 2 min, then incubated with the following antibodies: mouse anti-rat β-III tubulin monoclonal antibody (1:500; Cat.No. 05–559, Millipore, Billerica, MA, USA), rabbit anti-rat p-CREB (Ser133) monoclonal antibody (1:800; Cat.No. 9198, Cell Signaling Technology, USA), rabbit anti-rat CaMKK monoclonal antibody (1:250; Cat.No. ab174289, Abcam, USA), rabbit anti-rat CaMKI monoclonal antibody (1:250; Cat.No. ab68234, Abcam, USA), and rabbit anti-rat CaMKIV monoclonal antibody (1:250; Cat.No. ab75874, Abcam, USA) at 4°C overnight. The negative control omitted the primary p-CREB antibody. On the next day, the neurons were incubated with the secondary goat anti-mouse IgG labeled with cy3 (1:500, Cat.No. ab97035, Abcam, USA) [23] and goat anti-rabbit IgG labeled with cy2 (1:500, Cat.No. ab6940, Abcam, USA) at room temperature for 1 hr. Neuronal nuclei were stained with 20 μg/mL 4’,6-diamidino-2-phenylindole dihydrochloride (DAPI). Finally, neurons were observed with a fluorescence microscope (BX51, Olympus, Japan).

### Measurement of intracellular Ca^2+^ in DRGNs

Intracellular Ca^2+^ in DRGNs was measured as previously described [5]. During the initial 5 min of the experiment after subtracting background fluorescence, baseline fluorescence (F_0_) was measured as an average normalized fluorescence emitted. Ca^2+^ intensity was captured as fluorescence (F) from selected areas within a cell following the subtraction of background fluorescence. Fluorescence traces represented the fold fluorescence over baseline after background subtraction (F/F_0_). Cells were first incubated with drugs at 37°C in 5% CO_2_ for 30 min, washed in Hanks’ balanced salt solution (HBSS) twice, and then loaded with fluo-4 as previously described [5, 24].

### Lentiviral vector transduction in DRGNs

The lentivarial CREB-shRNA or CaMKII-shRNA with puromycin resistance (Genechem, Shanghai, China) was applied to specifically knockdown the transcription of CREB or CaMKII. Randomly chosen nonsense sequences were used as controls. The validated shRNA sequences were CREB: 5’-AAGCACTTAAGGACCTTTACT-3’ (GenBank NM 031017), CaMKIIα 5’-CCACTACCTTATCTTCGAT-3’ (GenBank NM 012920), and CaMKIIβ: 5’-GAGTATGCAGCTAAGATCA-3’ (GenBank NM 021739). Lentiviral vector transduction was carried out according to the procedure recommended by the manufacturer. Neurons were transduced at 20× infectious units in the presence of polybrene (8 μg/ml) at 37°C. After 24 hr, the supernatant was replaced with complete medium supplemented with puromycin. Following puromycin screening for 48 hr, transduced cells were treated with ES and collected for immunoblot, quantitative (q) PCR, or neurite length analyses. The down-regulation of protein expression of CREB, CaMKIIα and CaMKIIβ was determined by Western blot.

### Western blot

Neurons were collected and lysed in a mixture of 50 mM Tris-HCl (pH 8.0), 400 μM phenylmethylsulfonyl fluoride, 1.5 μM aprotinin, 0.5 M NaCl, 2 μM pepstatin, 4 μM leupeptin, and 0.5% Triton X-100. Neurons were lysed by homogenization and sonication. Forty micrograms of each protein sample were used for the assay. Denatured proteins were separated by Tris-glycine SDS-PAGE (Bio-Rad, USA). Separated proteins in gels were then transferred onto nitrocellulose membranes and were incubated with primary antibodies to CREB (rabbit monoclonal, 1:1000; Cat.No. 9197, Cell Signaling Technology, USA), p-CREB (pSer-133) (rabbit monoclonal, 1:1000; Cat.No. 9198, Cell Signaling Technology, USA), histone H1 (mouse monoclonal, 1:1000; Cat.No. ab11080, Abcam, USA), CaMKIIα (mouse monoclonal, 1:200; Cat.No. sc-13141, Santa Cruz Biotechnology, Inc., USA), CaMKIIβ (goat polyclonal, 1:200; Cat.No. sc-1540, Santa Cruz Biotechnology, Inc., USA), CaMKK (rabbit monoclonal, 1:1000; Cat.No. ab174289, Abcam, USA), CaMKI (rabbit monoclonal, 1:5000; Cat.No. ab68234, Abcam, USA), CaMKIV (rabbit monoclonal, 1:2000; Cat.No. ab75874, Abcam, USA), β-actin (rabbit polyclonal, 1:500; Cat.No. sc-130656, Santa Cruz Biotechnology, Inc., USA), and β-tubulin (mouse monoclonal, 1:500; Cat.No. sc-23949, Santa Cruz Biotechnology, Inc., USA) in 0.01 M PBS (pH 7.5) mixed with 0.1% Tween 20 and 5% skin-milk at 4°C overnight. Protein bands were visualized by an enhanced chemiluminescence kit (Thermo Scientific, USA) and film development. Protein bands were analyzed using ImageJ software (NIH, Bethesda, MD).

### Real-time qPCR analysis

Total RNA from treated neurons was extracted using RNAiso reagent (TaKaRa, Dalian, China). Total RNA (1 μg) of each group of cells was reverse-transcribed to cDNA using the reagent of PrimeScript^®^ RT Master Mix Perfect Real Time (TaKaRa, Dalian, China). The primers were as follows: β-actin (150 bp): 5’-GGAGATTACTGCCCTGGCTCCTA-3’ (forward), 5’-GACTCATCGTACTCCTGCTTGCTG-3’ (reverse); BDNF (89 bp): 5’-CAAAAGGCACTGGAACTCGC-3’ (forward), 5’-ACCGCCAGCCAATTCTCTTT-3’ (reverse). β-actin was used as loading control. qPCR was carried out using Mini Opticon^TM^ System (Bio-Rad, USA) with triplicates in 96-well plates. Each reaction (25 μL) consisted of 12.5 μL SYBR^®^ Premix Ex Taq^TM^ II (TaKaRa, Dalian, China), 2 μL template, 1 μL 10 μmol/L sense, and 1 μL 10 μmol/L anti-sense primers. The SYBR Green I Dye fluorescence in each cycle was monitored using Opticon Monitor^TM^ software (version 3.1, Bio-Rad). The data was analyzed according to the method described previously [25]. Levels of BDNF mRNA were normalized to that of β-actin mRNA.

### Determination of intracellular BDNF level

To confirm the changes of BDNF mRNA levels, intracellular amounts of BDNF protein were determined. Neurons were lysed using NP40 Cell Lysis Buffer (Thermo Scientific, USA). The protein concentration was determined by the Bradford assay. Rat BDNF enzyme linked immunosorbent assay (ELISA) kit (Thermo Scientific, USA) was used to analyze the changes of BDNF protein levels according to the protocol. Briefly, protein samples were added to wells pre-coated with biotinylated BDNF antibody. Streptavidin-HRP solution was added to each well and incubated for 45 min at room temperature with gentle shaking. Then, wells were washed and incubated with tetramethylbenzidine for 30 min at room temperature. The plate was evaluated within 30 min of stopping the reaction. Absorbance was measured on an ELISA plate reader set at 450 nm in a micro-plate reader (SUNOSTIK, China).

### Analysis of neurite length

A total of 8–10 random 10× or 20× fields were imaged using BX-51 fluorescence microscopes (Olympus, Tokyo, Japan). Neurite length analysis was carried out according to the method described in previous studies [5, 26, 27]. We used the basic criteria for assessing neurite length: only the maximal distance along a neurite was defined as the neurite length; if there was more than one neurite, the distance from the soma to the end of the longest neurite was adopted; if there were branched neurites, the distance from the soma to the end of the longest branch at each branch-point was adopted. The average neurite length per neuron in each group was calculated and compared.

### Drugs

Omega-conotoxin GVIA (CON) was used at a final concentration of 1 μM (dissolved in sterile distilled water). Dantrolene (DAN), thapsigargin (THA), KN93, KN62, and STO609 were used at final concentrations of 3, 5, 10, and 50 μM (dissolved in dimethyl sulfoxide (DMSO)). Nimodipine (NIM) was used at a final concentration of 2 μM (dissolved in ethanol). The solvents and optimized final concentrations were adopted from previous studies [5, 24]. Recombinant BDNF and hyperforin (HF) (Sigma, St Louis, MO, USA) were used at final concentrations of 10 ng/ml and 0.3 μM, respectively. According to previous studies [28, 29], the maximal concentration of ethanol or DMSO used was controlled at 0.02% and 0.1%, respectively.

### Cell viability assay

Neurons were first treated with drugs at 37°C in 5% CO_2_ for 30 min and washed twice with HBSS. Cell viability was evaluated with the staining of Hoechst 33342/propidium iodide (PI) according to a previously described protocol [24]. The calculation of cell viability was: Cell viability = 1 - (PI-positive cells/Hoechst 33342- positive cells × 100%).

### Data analysis

The results from several cultures (n ≥ 3) were pooled for statistical analysis. Data are shown as mean ± standard deviation (SD). Analysis of variance (ANOVA) with Tukey’s post-hoc multiple comparisons was used for the comparison of the ratios among groups (SPSS 13.0 software, Chicago, IL, USA). P < 0.05 was considered significantly different.

## Results

### ES induces p-CREB in DRGNs

**Fig 1:**
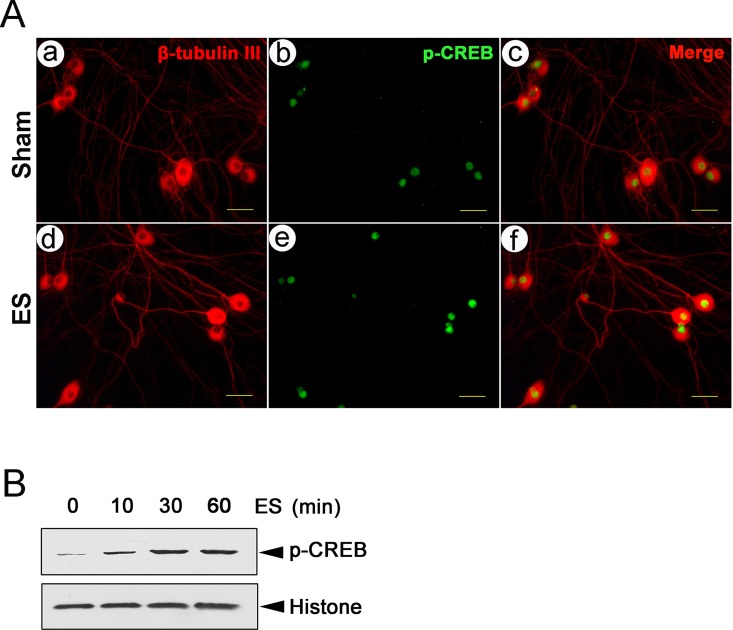
ES induces p-CREB in DRGNs. (A) p-CREB localization in cultured DRGNs with or without ES. p-CREB (green) was localized in the nuclei. β-tubulin III (red) indicated cell bodies and neurites. Double staining was performed in Shams (a, b, c) and cells with 30 min ES (d, e, f). Scale bar (50 μm) applies to a—f. (B) p-CREB expression in cells with 0–60 min ES (mean ± SD; n = 3). Histone expression was used as a loading control.

To address whether ES induces p-CREB, we used an antibody specific to p-CREB (pSer-133) for detection. p-CREB was localized in the nuclei of cultured DRGNs. In contrast, β-tubulin III was predominantly expressed in the cytosol and neurite (Fig 1A). Results in Fig 1B show that the p-CREB level was elevated in the ES group (5 V, 10 Hz, 30 min) compared to the control. 10 min ES was sufficient to induce elevation of p-CREB (P < 0.05); 30 min ES induced an approximate 5-fold increase in p-CREB compared to control (P < 0.05); and 60 min ES induced an increase in p-CREB level similar to 30 min ES (P > 0.05) (Fig 1B). Therefore, 10–60 min ES induced p-CREB in cultured DRGNs.

### Blocking intracellular Ca^2+^ suppresses ES-induced elevation of p-CREB

**Fig 2:**
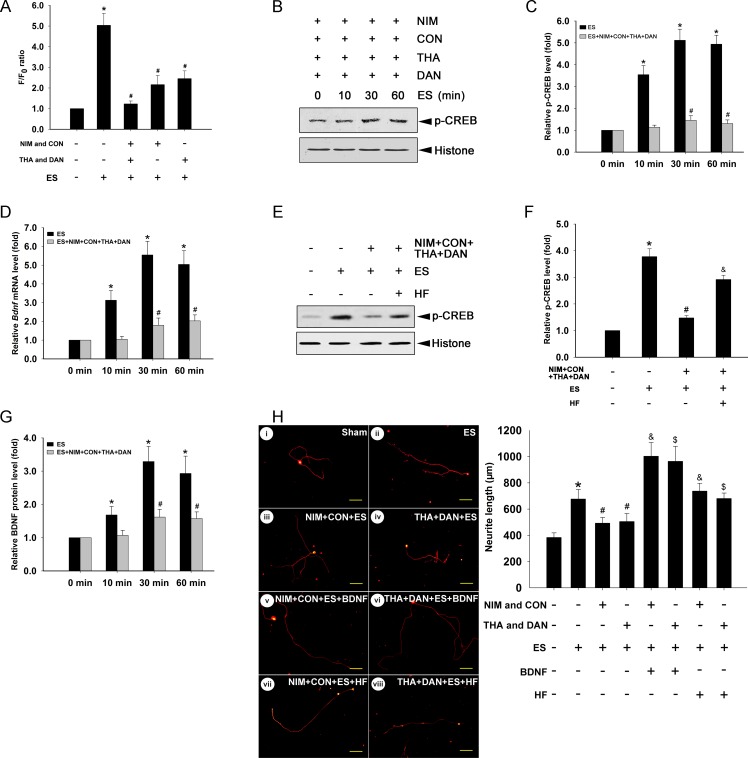
Blockade of intracellular Ca^2+^ suppresses ES-induced p-CREB mediated BDNF transcription and neurite outgrowth. (A) Relative Ca^2+^ levels in cells treated with Ca^2+^ inhibitors for 30 min and ES for 30 min (mean ± SD; n = 4). *P < 0.05 compared to sham; ^#^P < 0.05 compared to ES; *F*-value = 75.949. (B) p-CREB expression in cells treated with Ca^2+^ inhibitors and ES (mean ± SD; n = 3). Histone expression was used as a loading control. (C) Relative p-CREB levels in cells treated with ES (*F*-value = 72.756) or ES + Ca^2+^ inhibitors (*F*-value = 5.649) (mean ± SD; n = 3). *P < 0.001 compared to 0 min ES group; ^#^P < 0.05 compared to group with 0 min ES + Ca^2+^ inhibitors. (D) Relative BDNF mRNA levels in cells treated with ES (*F*-value = 41.627) or ES + Ca^2+^ inhibitors (*F*-value = 12.051) (mean ± SD; n = 4). β-actin was used as a loading control. *P < 0.05 compared to 0 min ES group; ^#^P < 0.05 compared to group with 0 min ES + Ca^2+^ inhibitors. (E) p-CREB expression in cells treated with Ca^2+^ inhibitors, ES, and HF. Histone expression was used as a loading control. (F) Quantification of p-CREB treated with Ca^2+^ inhibitors, ES, and HF (mean ± SD; n = 3). *P < 0.05 compared to Sham group; ^#^P < 0.05 compared to ES+; ^&^P < 0.05 compared to ES+/Ca^2+^ inhibitors; *F*-value = 28.325. (G) Relative BDNF protein levels in cells treated with ES (*F*-value = 22.937) or ES + Ca^2+^ inhibitors (*F*-value = 10.186) (mean ± SD; n = 4). *P < 0.05 compared to 0 min ES group; ^#^P < 0.05 compared to group with 0 min ES + Ca^2+^ inhibitors. (H) Typical images of neurite outgrowth in Sham and cells with treatments. β-tubulin III was used to indicate cell bodies and neuritis. Scale bar (100 μm) applies to i—viii. Quantification of neurite outgrowth in treated cells (mean ± SD; n = 3). Numbers of cell neurites analyzed in each group: (i) 108, (ii) 97, (iii) 112, (iv) 120, (v) 105, (vi) 120, (vii) 117, (viii) 101. *P < 0.001 compared to Sham group; ^#^P < 0.05 compared to ES group; ^&^P < 0.05 compared to ES+/NIM and CON group; ^$^P < 0.05 compared to ES+/THA and DAN group; *F*-value = 17.125; NIM, nimodipine; CON, omega (ω)-conodoxin-GVIA; THA, thapsigargin; DAN, dantrolene; ES, electrical stimulation; HF, hyperforin.

We previously showed that ES increases intracellular Ca^2+^ in cultured DRGNs [5]. To examine whether Ca^2+^ is involved in ES-induced p-CREB, we applied Ca^2+^ inhibitors to the cell cultures and analyzed the levels of p-CREB after ES. 30 min ES significantly increased intracellular Ca^2+^ (P < 0.05, compared to the sham group). Application of voltage-gated Ca^2+^ channel blockers NIM (2 μM) and CON (1 μM), and Ca^2+^ store inhibitors DAN (50 μM) and THA (10 μM) for 30 min, followed by ES 30 min, completely blocked the Ca^2+^ increase (P < 0.05, compared to the ES group). Application of NIM (2 μM) and CON (1 μM) for 30 min, or DAN (50 μM) and THA (10 μM) for 30 min, followed by ES for 30 min, also significantly blocked the Ca^2+^ increase compared to the ES group (P < 0.05 for both comparisons) (Fig 2A). Treatment of four or two Ca^2+^ inhibitors for 30 min had no effect on cell viability (data not shown). Hence, the application of inhibitors for either a single or two types of Ca^2+^ channels sufficiently blocked ES-induced Ca^2+^ increases in DRGNs.

The p-CREB levels were examined in cells treated with Ca^2+^ inhibitors and ES. Treatment with four inhibitors for 30 min followed by ES for 10 min did not induce a significant increase in p-CREB. Treatment with four inhibitors for 30 min, and ES for 30 or 60 min, increased p-CREB compared to the control group (with four inhibitors for 30 min and ES for 0 min) (P < 0.05 for both comparisons) (Fig 2B and 2C). However, compared to groups without Ca^2+^ inhibitors, the 10, 30 or 60 min ES induced p-CREB increase was greatly suppressed (Fig 2C). Therefore, treatment with four Ca^2+^ inhibitors for 30 min effectively suppressed p-CREB.

### Blocking intracellular Ca^2+^ suppresses ES-induced BDNF transcription and neurite outgrowth

We next examined the effect of Ca^2+^ blockade on ES-induced BDNF transcription and neurite outgrowth. Cultured cells were incubated with four Ca^2+^ inhibitors for 30 min and subjected to ES. The BDNF mRNA level in each group was normalized to the β-actin mRNA level (loading control) and then normalized to the 0 min control group. Without treatment of Ca^2+^ inhibitors, 10 min ES increased BDNF transcription approximately 3.1-fold relative to control (P < 0.05); 30 min ES increased BDNF transcription approximately 5.5-fold (P < 0.05); and ES for 60 min increased BDNF transcription approximately 5.0-fold (P < 0.05) (Fig 2D). Treatment with the four Ca^2+^ inhibitors for 30 min did not significantly increase BDNF transcription in response to 10 or 30 min ES, but there was a moderate increase in BDNF transcription (~2-fold) after 60 min ES compared to 0 min ES (P < 0.05) (Fig 2D). Thus, application of Ca^2+^ inhibitors for 30 min suppressed BDNF transcription in DRGNs. The changes in BDNF mRNA levels were consistent with the changes in protein levels (Fig 2G).

The effect of Ca^2+^ inhibitors on ES-induced neurite outgrowth was examined. ES for 30 min increased neurite length compared to control (P < 0.05) (Fig 2H). Treatment with voltage-gated Ca^2+^ channel blockers (NIM and CON) for 30 min, followed by 30 min of ES, blocked neurite outgrowth compared to the ES group (P < 0.05) (Fig 2H). Similarly, treatment with Ca^2+^ store inhibitors (THA and DAN) for 30 min, followed by 30 min ES, blocked neurite outgrowth compared to the ES group (P < 0.05) (Fig 2H). Thus, the application of Ca^2+^ inhibitors for 30 min blocked ES-induced neurite outgrowth in cultured DRGNs.

In order to determine whether the effects of Ca^2+^ inhibitors can be reversed by BDNF application or constitutively active CREB, recombinant BDNF [30–32] or HF was added into the primary neuron culture medium after ES. HF has been shown to increase phosphorylation of CREB *in vivo* [33] and *in vitro* [34–36]. Data showed that the p-CREB level in the Ca^2+^ inhibitors/ES/HF group was increased approximately 2-fold by HF compared to that in the Ca^2+^ inhibitors/ES group (P < 0.05) (Fig 2E and 2F). HF (0.3 μM) also increased neurite outgrowth in the Ca^2+^ inhibitors/ES/HF group (P < 0.05, compared to the Ca^2+^ inhibitors/ES group) (Fig 2H). Furthermore, the inhibitory effect of Ca^2+^ inhibitors on neurite outgrowth was reversed by BDNF (10 ng/ml; Sigma, St Louis, MO, USA) (P < 0.05, compared to the Ca^2+^ inhibitors/ES group) (Fig 2H).

### p-CREB is required for ES-induced BDNF transcription and neurite outgrowth

**Fig 3:**
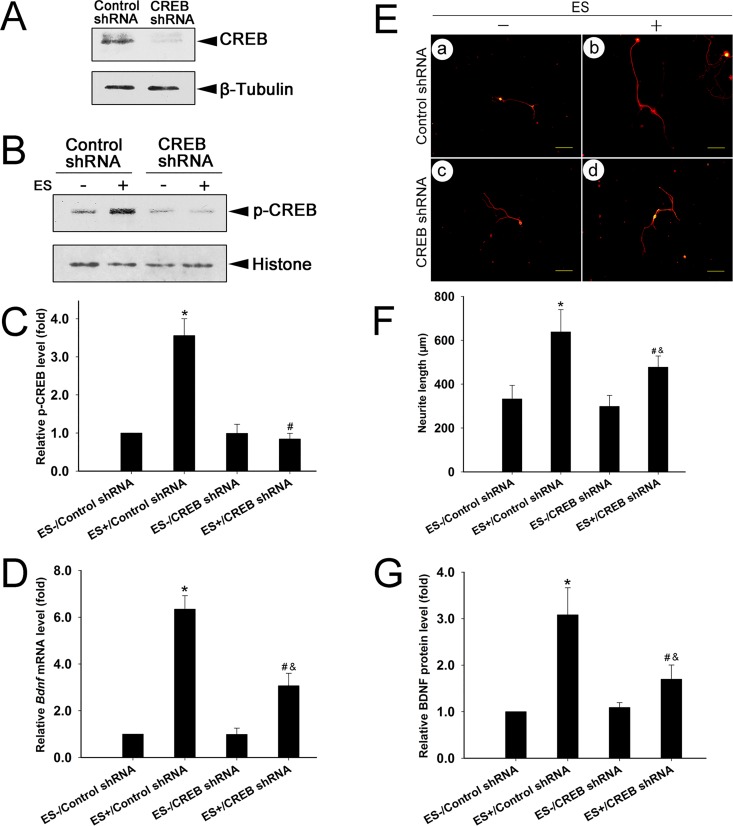
p-CREB is involved in ES-induced BDNF mRNA transcription and neurite outgrowth. (A) CREB expression in cells transduced with control or CREB shRNA (mean ± SD; n = 3). β-tubulin was used as a loading control. (B, C) p-CREB expression in transduced cells with or without ES (mean ± SD; n = 3). Histone was used as a loading control. *P < 0.001 compared to ES-/Control shRNA; ^#^P < 0.001 compared to ES+/Control shRNA; *F*-value = 49.293. (D) Relative BDNF mRNA levels in cells of the four groups (B) (mean ± SD; n = 4). β-actin was used as a loading control. *P < 0.001 compared to ES-/Control shRNA; ^#^P < 0.05 compared to ES+/Control shRNA; ^&^P < 0.05 compared to ES-/CREB shRNA; *F*-value = 37.889. (E) Typical images of neurite outgrowth in transduced cells with or without ES. β-tubulin III was used to indicate cell bodies and neuritis. Scale bar (100 μm) applies to a—d. (F) Quantification of neurite outgrowth in treated cells (E) (mean ± SD; n = 3). The assay was performed in triplicate. Numbers of cell neurites analyzed in each group: (a) 114, (b) 108, (c) 117, (d) 96. *P < 0.05 to ES-/Control shRNA; ^#^P < 0.05 compared to ES+/Control shRNA; ^&^P < 0.05 compared to ES-/CREB shRNA; *F*-value = 10.190. (G) Relative BDNF protein levels in cells of the four groups as (B) (mean ± SD; n = 4). *P < 0.001 compared to ES-/Control shRNA; ^#^P < 0.05 compared to ES+/Control shRNA; ^&^P < 0.05 compared to ES-/CREB shRNA; *F*-value = 20.396.

To assess whether p-CREB is involved in ES-induced BDNF transcription, we knocked down CREB by transducing cells with lentiviral shRNA targeting CREB. The DRGNs were incubated with media containing control or lentiviral shRNA targeting CREB for 24 hr and subjected to puromycin screening. The successfully transduced cells were resistant to puromycin and were selected for 30 min ES. Data showed that the expression of endogenous CREB was strongly suppressed by CREB shRNA compared to control shRNA (Fig 3A). 30 min ES increased p-CREB in the cells transduced with control shRNA (P < 0.001, compared to cells without ES), whereas the ES-induced p-CREB elevation was blocked in the cells transduced with CREB shRNA (P < 0.001, compared to cells with control shRNA and 30 min ES) (Fig 3B and 3C). Therefore, CREB shRNA specifically blocked ES-induced p-CREB.

The effect of p-CREB knockdown on ES-induced BDNF transcription was examined. 30 min ES increased BDNF transcription in cells transduced with control shRNA (P < 0.05). The BDNF transcription was also increased by 30 min ES in cells transduced with CREB shRNA compared to cells without ES (P < 0.05). However, the BDNF mRNA level was significantly lower in cells transduced with CREB shRNA compared to control shRNA (P < 0.05) (Fig 3D). The changes in BDNF mRNA were consistent with the changes in protein expression (Fig 2G).

The effect of p-CREB knockdown on ES-induced neurite outgrowth was also examined. 30 min ES increased neurite length in cells transduced with control shRNA (P < 0.05). 30 min ES also induced neurite outgrowth in cells transduced with CREB shRNA (P < 0.05). However, ES-induced neurite outgrowth was blocked in cells transduced with CREB shRNA compared to control shRNA (P < 0.05) (Fig 3E and 3F). Together, specific knockdown of p-CREB effectively blocked ES-induced BDNF transcription and neurite outgrowth in cultured DRGNs.

### Both CaMKIIα and CaMKIIβ subunits are required for ES-induced p-CREB

**Fig 4:**
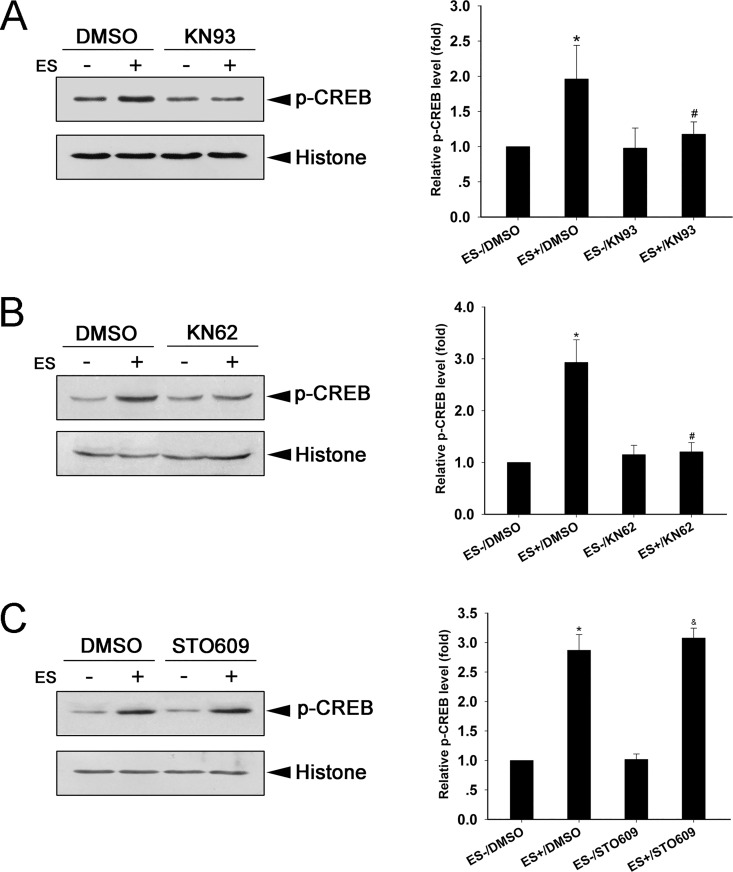
CaMKII is required for ES-induced p-CREB in DRGNs. Expression and quantification of p-CREB in cells treated with CaMKs inhibitors KN93 (A; *F*-value = 11.832), KN62 (B; *F*-value = 32.494), and STO609 (C; *F*-value = 18.096) (mean ± SD; n = 3). Histone was used as a loading control. DMSO was used as vehicle control. *P < 0.05 compared to ES-/DMSO group; ^#^P < 0.05 compared to ES+/DMSO group; ^&^P < 0.05 compared to ES-/STO609.

**Fig 5:**
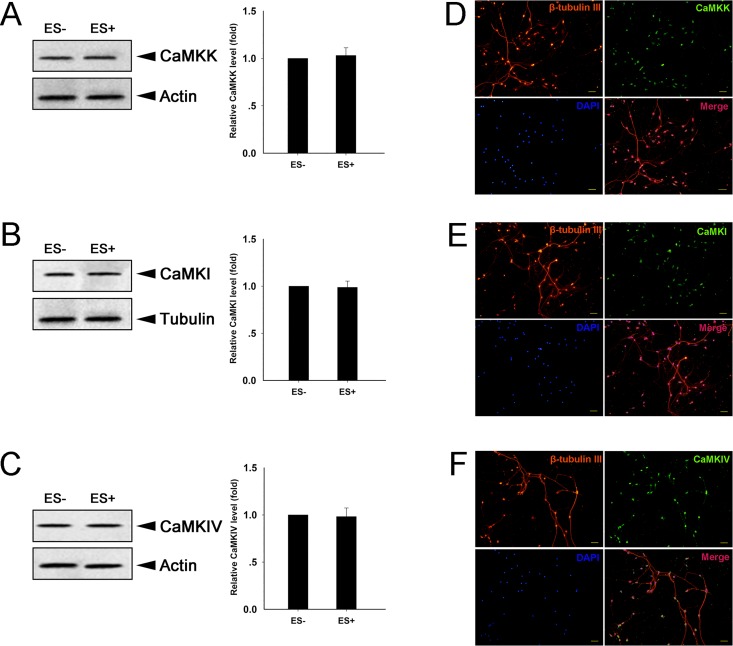
Expression confirmation of CaMK family members in DRGNs. Assessment and quantification of CaMKK (A; *F*-value = 11.832), CaMKI (B; *F*-value = 32.494), and CaMKIV (C; *F*-value = 18.096) by Western blot (mean ± SD; n = 3). Beta-actin or β-tubulin was used as the loading control. *P < 0.05 compared to ES- group. Expression of CaMKK (D), CaMKI (E), and CaMKIV (F) in DRGNs by immunostaining assay. Scale bar = 100 μm.

The CaMK family is critical for gene transcription, cell survival and death [18, 19, 37–39]. We treated culture DRGNs with either KN93 (CaMKII inhibitor), KN62 (CaMKII inhibitor), or STO609 (inhibitor of CaMKK, which activates CaMKI and CaMKIV but not CaMKII) to determine which CaMK isoform is tightly involved in ES-induced p-CREB. Cultured DRGNs were treated with CaMKs inhibitor for 30 min and subjected to ES for 30 min. The ES-induced p-CREB was blocked in cells treated with KN93 compared to cells treated with vehicle control (DMSO) (P < 0.05) (Fig 4A). Similarly, the ES-induced p-CREB was strongly blocked in cells treated with KN62 compared to cells treated with DMSO (P < 0.05) (Fig 4B). The ES-induced p-CREB was not affected in cells treated with STO609 compared to cells treated with DMSO (Fig 4C). We also detected the expressions of other CaMK family members (CaMKK/CaMKI/CaMKIV) in DRGNs. Western blot and immunostaining were used to assess the expression of these proteins in culture DRGNs. Western blot analysis showed that there were no differences between the protein levels of CaMKK/CaMKI/CaMKIV prior to and after ES conditions (Fig 5A–5C), and immunostaining assay showed that the DRGNs used in this study do express CaMKK, CaMKI, and CaMKIV (Fig 5D–5F).

The above results indicate that CaMKII, but not CaMKK, is involved in ES-induced p-CREB.

**Fig 6:**
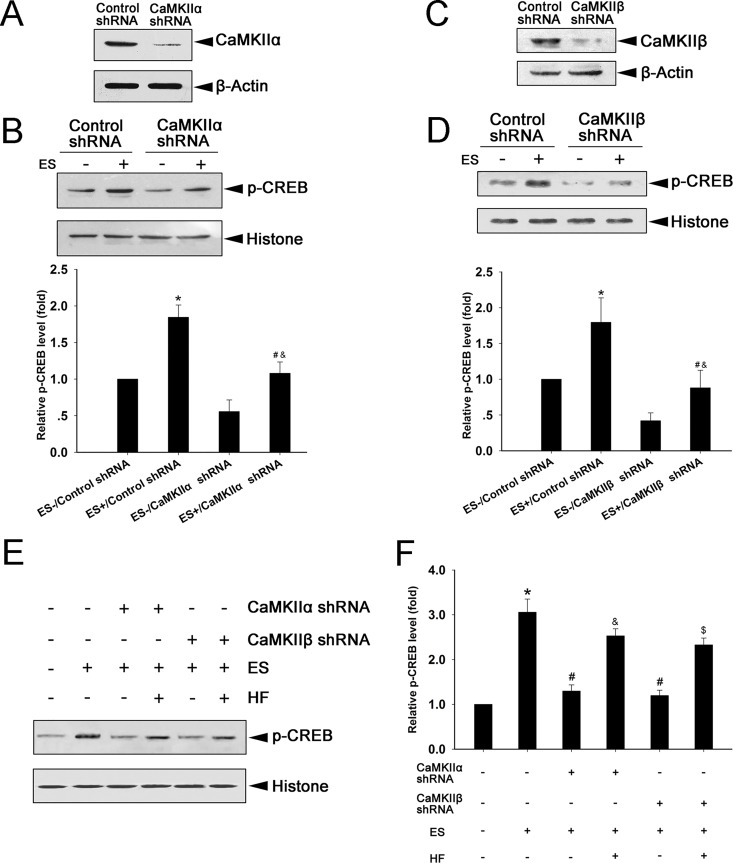
CaMKIIα and CaMKIIβ subunits are both required for ES-induced p-CREB. (A) Expression of CaMKIIα in cells transduced with control or CaMKIIα shRNA (mean ± SD; n = 3). β-actin was used as a loading control. (B) Expression and quantification of p-CREB in treated cells (mean ± SD; n = 3). Histone was used as loading control. *P < 0.05 compared to ES-/Control shRNA; ^#^P < 0.05 compared to ES+/Control shRNA; ^&^P < 0.05 compared to ES-/CaMKIIα shRNA; *F*-value = 24.440. (C) Expression of CaMKIIβ in cells transduced with control or CaMKIIβ shRNA (mean ± SD; n = 3). β-actin was used as a loading control. (D) Expression and quantification of p-CREB in treated cells (mean ± SD; n = 3). Histone was used as loading control. *P < 0.05 compared to ES-/Control shRNA; ^#^P < 0.05 compared to ES+/Control shRNA; ^&^P < 0.05 compared to ES-/CaMKIIβ shRNA; *F*-value = 24.173. (E) p-CREB expression in treated cells. Histone expression was used as a loading control. (F) Quantification of p-CREB treated cells (mean ± SD; n = 3). *P < 0.05 compared to sham group; ^#^P < 0.05 compared to ES+; ^&^P < 0.05 compared to ES+/CaMKIIα shRNA; ^$^P < 0.05 compared to ES+/CaMKIIβ shRNA; *F*-value = 28.325.

Studies have shown that CaMKII is involved in the regulation of gene expression and dendrite growth [40, 41]. Multiple isoforms of CaMKII have been identified in eukaryotes [42]. To further explore the involvement of CaMKII in the ES-induced p-CREB signaling, specific knockdown of the CaMKIIα or CaMKIIβ subunit by lentiviral shRNA in cultured cells was performed, and the effect on ES-induced p-CREB was measured. Expression of CaMKIIα was potently suppressed in cells transduced with CaMKIIα shRNA compared to control shRNA (Fig 6A). In cells transduced with control shRNA, p-CREB was increased by 30 min ES compared to 0 min ES (P < 0.05). In cells transduced with CaMKIIα shRNA, p-CREB was also increased by 30 min ES compared with 0 min ES (P < 0.05). However, ES-induced p-CREB was significantly lower in cells transduced with CaMKIIα shRNA compared to control shRNA (P < 0.05) (Fig 6B). Similarly, CaMKIIβ shRNA strongly suppressed CaMKIIβ expression (Fig 6C). The ES-induced p-CREB was significantly suppressed by the transduction of CaMKIIβ shRNA compared to control shRNA (P < 0.05); although 30 min ES was still able to induce p-CREB in cells transduced with CaMKIIβ shRNA (P < 0.05) (Fig 6D). HF (0.3 μM) was used to examine whether the suppression of p-CREB induced by CaMKII knockdown can be reversed. Data showed that HF significantly up-regulated the level of p-CREB compared to knockdown of CaMKIIα or CaMKIIβ (P < 0.05). These results indicate that both CaMKIIα and CaMKIIβ are required for ES-induced p-CREB.

### CaMKII and CaMKK are both required for ES-induced neurite outgrowth

**Fig 7:**
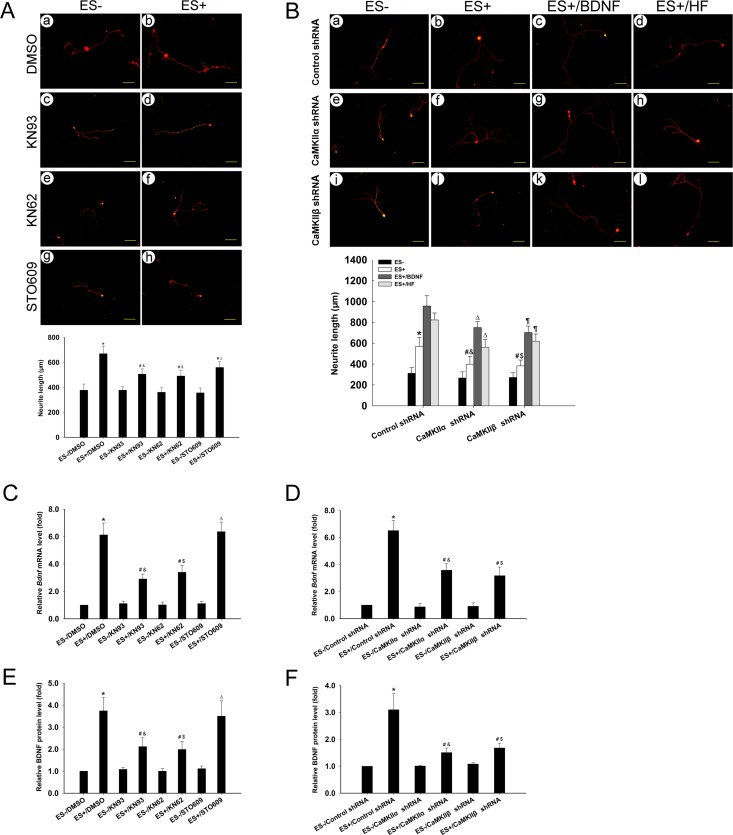
CaMKII (CaMKIIα and CaMKIIβ subunits) is required for ES-induced neurite outgrowth and BDNF mRNA transcription in DRGNs. (A) Neurite outgrowth in cells treated with KN93, KN62, or STO609 (mean ± SD; n = 3). β-tubulin III was used to indicate cell bodies and neurites. The assay was performed in triplicate. Numbers of cell neurites analyzed in each group: (a) 96, (b) 106, (c) 95, (d) 108, (e) 110, (f) 97, (g) 99, (h) 103. *P < 0.001 compared to ES-/DMSO group; ^#^P < 0.05 compared to ES+/DMSO group; ^&^P < 0.05 compared to ES-/KN93 group; ^$^P < 0.05 compared to ES-/KN62 group; ^∆^P < 0.05 compared to ES-/STO609 group; *F*-value = 15.362; Scale bar: 100 μm. (B) Neurite outgrowth in cells treated with CaMKIIα shRNA, CaMKIIβ shRNA and control shRNA (mean ± SD; n = 3). β-tubulin III was used to indicate cell bodies and neurites. The assay was performed in triplicate. Numbers of cell neurites analyzed in each group: (a) 92, (b) 90, (c) 95, (d) 101, (e) 97, (f) 93, (g) 98, (h) 94, (i) 91, (j) 95, (k) 93, (l) 102. *P < 0.05 compared to ES-/Control shRNA; ^#^P < 0.05 compared to ES+/Control shRNA; ^&^P < 0.05 compared to ES-/CaMKIIα shRNA; ^∆^P < 0.05 compared to ES+/CaMKIIα shRNA; ^$^P < 0.05 compared to ES-/CaMKIIβ shRNA; ^¶^P < 0.05 compared to ES+/CaMKIIβ shRNA; *F*-value = 12.624; Scale bar: 100 μm. (C) Relative BDNF mRNA levels in cells of the eight groups (A) (mean ± SD; n = 4). β-actin was used as a loading control. *P < 0.001 compared to ES-/DMSO group; ^#^P < 0.05 compared to ES+/DMSO group; ^&^P < 0.05 compared to ES-/KN93 group; ^$^P < 0.05 compared to ES-/KN62 group; ^∆^P < 0.05 compared to ES-/STO609 group; *F*-value = 49.792. (D) Relative BDNF mRNA levels in cells of the eight groups (B) (mean ± SD; n = 4). β-actin was used as a loading control. *P < 0.001 compared to ES-/Control shRNA; ^#^P < 0.05 compared to ES+/Control shRNA; ^&^P < 0.05 compared to ES-/CaMKIIα shRNA; ^$^P < 0.05 compared to ES-/CaMKIIβ shRNA; *F*-value = 36.082. (E) Relative BDNF protein levels in cells of the eight groups (A) (mean ± SD; n = 4). *P < 0.001 compared to ES-/DMSO group; ^#^P < 0.05 compared to ES+/DMSO group; ^&^P < 0.05 compared to ES-/KN93 group; ^$^P < 0.05 compared to ES-/KN62 group; ^∆^P < 0.05 compared to ES-/STO609 group; *F*-value = 27.532. (F) Relative BDNF protein levels in cells of the eight groups (B) (mean ± SD; n = 4). *P < 0.001 compared to ES-/Control shRNA; ^#^P < 0.05 compared to ES+/Control shRNA; ^&^P < 0.05 compared to ES-/CaMKIIα shRNA; ^$^P < 0.05 compared to ES-/CaMKIIβ shRNA; *F*-value = 19.796.

We further examined the contributions of CaMKs to ES-induced neurite outgrowth in cultured DRGNs. When DRGNs were pretreated with KN93 or KN62, ES-induced neurite outgrowth was markedly suppressed (P < 0.05) (Fig 7A). After incubation with STO609, ES-induced neurite outgrowth was also suppressed in DRGNs compared to the ES+/DMSO group (P < 0.05) (Fig 7A). The neurite length increased in the ES+/KN93, ES+/KN62, and ES+/STO609 groups compared to the the ES-/KN93, ES-/KN62, and ES-/STO609 groups (P < 0.05) (Fig 7A). The results showed that CaMKII and CaMKK were both required for ES-induced neurite outgrowth in DRGNs.

The effect of CaMKIIα or CaMKIIβ knockdown on neurite outgrowth in DRGNs was analyzed. Knockdown of CaMKIIα or CaMKIIβ partially blocked ES-induced neurite outgrowth (P < 0.05) (Fig 7B). Application of BDNF (10 ng/ml) and HF (0.3 μM) both reversed the inhibition on neurite outgrowth induced by CaMKIIα and CaMKIIβ (P < 0.05) (Fig 7B). Moreover, ES induced neurite outgrowth in the ES+/CaMKIIα shRNA or CaMKIIβ group compared to the ES-/CaMKIIα shRNA or ES-/CaMKIIβ shRNA group, separately (P < 0.05) (Fig 7B). These results suggest that CaMKIIα and CaMKIIβ are both required for ES-induced neurite outgrowth in DRGNs.

### Both CaMKIIα and CaMKIIβ are required for ES-induced BDNF mRNA transcription

KN93, KN62 and STO609 were used to determine which CaMK isoform is involved in ES-induced BDNF mRNA transcription and neurite outgrowth. When DRGNs were treated with KN93 or KN62, ES-induced BDNF mRNA transcription was suppressed (P < 0.05) (Fig 7C). After incubation with STO609, ES-induced BDNF mRNA transcription was unaffected (P > 0.05) (Fig 7C). Treatment of cells with the combination of ES and KN93 or KN62 promoted ES-induced BDNF mRNA transcription (P < 0.05) (Fig 7C) compared to KN93 or KN62 treatment only. The results showed that CaMKII, but not CaMKK, was involved in ES-induced BDNF mRNA transcription.

We next tested which isoform of CaMKII is involved in BDNF mRNA transcription induced by ES. The qPCR analysis showed that both CaMKIIα shRNA and CaMKIIβ shRNA significantly blocked ES-induced BDNF mRNA transcription (P < 0.05) (Fig 7D). Moreover, the BDNF mRNA level was still up-regulated in either the ES+/CaMKIIα shRNA or ES+/CaMKIIβ shRNA group compared to the respective ES-/CaMKIIα shRNA or ES+/CaMKIIβ shRNA group (P < 0.05) (Fig 7D). The results indicate that CaMKIIα and CaMKIIβ play critical roles in ES-induced BDNF mRNA transcription in DRGNs. The changes in BDNF levels were consistent with the changes in BDNF mRNA levels (Fig 7E and 7F).

## Discussion

It has been established that ES induces BDNF transcription and neurite outgrowth accompanied by an elevation of intracellular Ca^2+^ in rat DRGNs [5]. It is unclear whether the Ca^2+^ elevation is involved in ES-induced BDNF transcription and neurite outgrowth. The current study aimed to identify a signaling pathway mediating ES-induced neuronal activation initiated by Ca^2+^ elevation. We showed that Ca^2+^ blockers inhibited ES-induced p-CREB, BDNF transcription, and neurite outgrowth, and that knockdown of CREB suppressed ES-induced neuronal activation. We further showed that CaMKII, specifically, both CaMKIIα and CaMKIIβ subunits, but not CaMKK, was required for ES-induced p-CREB. We propose that the novel signaling pathway of Ca^2+^-CaMKII-CREB plays a crucial role in ES-induced BDNF transcription and neurite outgrowth in cultured rat DRGNs.

ES is one of the most effective approaches for depolarizing the membrane potential of neurons and stimulating gene expression. ES treatment induces a rapid and dramatic Ca^2+^ increase in DRGNs [5], spinal cord neurons [43], PC12 cells [33], hippocampal neurons [44], and cultured retinal Müller cells [45]. ES increases Ca^2+^ levels and activates nerve growth factor in ES-responsive Schwann cells *in vitro* [28, 46]. Ca^2+^, a critical second messenger, is involved in many intracellular molecular signaling cascades [47, 48]. Previous studies have shown that Ca^2+^ plays important roles in regulating neurite extension triggered by extracellular factors, such an chemicals, neurotrophin or physical stimulation [5, 49, 50]. In the developing central nervous system (CNS), Ca^2+^ regulates neurite extension and retraction and axon guidance [51–53]. The signaling pathways that mediate Ca^2+^-dependent phosphorylation of CREB, which thereby regulates gene transcription, have been intensively investigated. Previous studies have demonstrated that there are other CREB regulators. It is reported that nerve growth factor (NGF)-regulated extracellular regulated kinase (ERK) phosphorylation triggers CREB phosphorylation, which recruits the CREB binding protein (CBP) to the NGF promoter regions of cAMP-responsive genes related to neuronal morphology change, synaptic plasticity, and dendritic spine growth [54]. In addition to ERK/mitogen-activated protein kinases (MAPKs), various signaling cascades can phosphorylate CREB, including CaMKs [55], phosphatidylinositol 3-kinase (PI3K)/Akt [56], cAMP-dependent protein kinase A (PKA) [57], and protein kinase C (PKC) [58].

One of the most important genes regulating neuronal activity is BDNF [59]. BDNF is involved in the regulation of axonal regeneration, dendritic outgrowth, and synaptic plasticity [60]. Studies show that BDNF combined with suboptimal levels of NGF suppresses the growth of sympathetic neurons to sprout neurites *in vitro* [61]. The outgrowth of rat superior cervical ganglion explants cultured with uterine horns of BDNF^-/-^ mice in the absence of estrogen (E_2_) is slowed compared to wild-type [62]. In transgenic mice over-expressing BDNF, axons avoid regions with high expression of BDNF protein [63]. The reduced BDNF, due to a mutation of the BDNF allele, indicates the absence of an inhibitory influence on sympathetic sprouting [61]. Therefore, BDNF is widely recognized as an important mediator of axon regrowth under the conditions of genetic modification.

Reduced CREB activity inhibits BDNF expression [8]. Given BDNF’s importance for nervous system development and function, the mechanisms by which Ca^2+^ regulates CREB and how CREB family members activate BDNF transcription and translation need to be further studied. It is critical to characterize the mechanisms by which BDNF mRNA levels regulate neuronal activity (e.g. neurite outgrowth or synaptic formation). In addition, calcium-responsive transcription factor (CaRF) is an important mediator for BDNF activity regulated by calcium influx in neurons; however, the detailed mechanism of CaRF and the relative signaling pathways are still undefined [64]. Another finding showed that 3,6’-Disinapoyl sucrose-triggered regulation of BDNF gene expression was associated with CREB-dependent BDNF transcription and upstream molecular activation of CaMKII and ERK1/2 in SH-SY5Y cells [10]. CREB is activated by the Ca^2+^ and CaMK pathway, specifically CaMKIV in the cortical neurons [8]. On the contrary, another study showed that CaMKII negatively regulated p-CREB and thus repressed BDNF expression through promoter region III [65].

CaMKII is one of the most critical regulators of axon regeneration and neurite regrowth [66, 67]. CaMKIIβ is more capable of promoting neurite outgrowth than CaMKIIα in mouse Nb2a neuroblastoma cells [67]. Saito et al. [68] found that knockdown of CaMKIIβ suppressed BDNF-induced neuritogenesis of cortical neurons in the rat at embryonic day 18, but knockdown of CaMKIIα did not affect neuritogenesis, indicating that there was difference in expression between CaMKIIα and CaMKIIβ in the early developmental stage of cortical neurons. However, studies report that CaMKII overexpression suppresses neurite regrowth induced by dibutyryl cyclic AMP or NGF [66, 69].

Ca^2+^-induced CaMK activation in primary cultured neurons triggers phosphorylation of CREB at Ser133, 142, and 143, which are all necessary for Ca^2+^-induced intracellular gene transcription [70]. It has been found that CREB phosphorylation at Ser142 and Ser143 contributes to CREB activation, and that mutations of Ser142 and Ser143 to alanines inhibits Ca^2+^-induced CREB-dependent transcription, while CREB phosphorylation at Ser142 alone reduced the transcriptional activation [65, 70]. However, data obtained from recent *in vivo* studies implies that the mechanism could be much more complicated. Gau et al. [71] developed transgenic mice with a CREB Ser142-to-Ala mutation, and found that the transgenic mice had a changed circadian clock, which may lead to down-regulation of c-Fos, a transcription factor regulated by CREB.

The effects of cAMP on transcription-dependent axon growth have been attributed to the activation of CREB [72]. To assess the role of CREB in neurite growth, Ma et al. [73] expressed a constitutively active CREB fusion protein (referred to as CREB-CA) and a dominant-negative CREB protein (referred to as CREB-DN). CREB-CA enhanced basal neurite growth on both MAG- and R2-CHO cells [72]. Interestingly, CREB-CA showed marked synergy with Bt_2_cAMP, causing a greater than additive increase in neurite growth on MAG-CHO cells. The Bt_2_cAMP-mediated neurite growth persisted in neurons expressing CREB-DN. These results imply that, although activation of CREB-dependent gene expression can by itself promote neurite growth, it is not necessary for cAMP-triggered growth, which signifies the existence of an alternative growth-promoting pathway downstream of cAMP.

**Fig 8:**
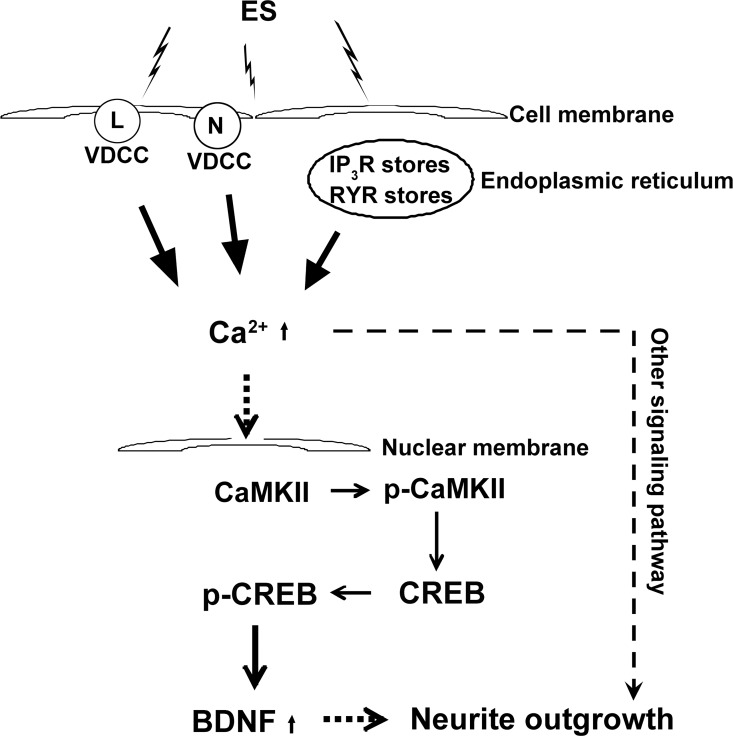
Potential scheme for the Ca^2+^-CaMKII-CREB signaling pathway that is involved in ES-induced neurite outgrowth and BDNF transcription.

The current findings together with a previous report [5] support our working hypothesis that ES induces an increase in [Ca^2+^]_*i*_ through Ca^2+^ influx through L- and N-type voltage-dependent calcium channels, and calcium mobilization from inositol triphosphate-3 receptor (IP3R)-sensitive calcium stores and ryanodine receptor (RYR)-sensitive calcium stores. The initial rise in [Ca^2+^]_*i*_ activates downstream CaMKII, which subsequently triggers CREB phosphorylation, thereby promoting BDNF gene expression and neurite outgrowth (schematic shown in Fig 8). These findings provide clues to further understand the pathophysiological mechanisms of nerve regeneration promoted by ES. Our findings indicate that ES has clinical relevance for improving functional recovery after nerve injury, although the mechanism of ES-induced nerve repair and regeneration still require further investigation. Additionally, this study indicates that CaMKK may be involved in ES-induced neurite outgrowth, possibly *via* a CREB-independent signaling pathway. Future studies will investigate the role of CaMKK in ES-induced neurite outgrowth in neuronal cells.
